# Biomarkers for Predicting Malignant Pleural Mesothelioma in a Mexican Population

**DOI:** 10.7150/ijms.23939

**Published:** 2018-06-04

**Authors:** Guadalupe Aguilar-Madrid, Beate Pesch, Emma S Calderón-Aranda, Katarzyna Burek, Carmina Jiménez-Ramírez, Cuauhtémoc Arturo Juárez-Pérez, María Dolores Ochoa-Vázquez, Luis Torre-Bouscoulet, Leonor Concepción Acosta-Saavedra, Isabel Sada-Ovalle, Jorge García-Figueroa, Isabel Alvarado-Cabrero, Patricia Castillo-González, Alejandra Renata Báez-Saldaña, José Rogelio Pérez-Padilla, Juvencio Osnaya-Juárez, Rosa María Rivera-Rosales, Eric Marco García-Bazán, Yolanda Lizbeth Bautista-Aragón, Elimelec Lazcano-Hernandez, Daniel Alejandro Munguía-Canales, Luis Marcelo Argote-Greene, Dirk Taeger, Daniel Gilbert Weber, Swaantje Casjens, Irina Raiko, Thomas Brüning, Georg Johnen

**Affiliations:** 1 Research Unit Health at Work, XXI Century National Medical Center (CMNSXXI), Instituto Mexicano del Seguro Social (IMSS), Mexico City, Mexico; 2 Institute for Prevention and Occupational Medicine of the German Social Accident Insurance, Institute of the Ruhr-University Bochum (IPA), Bochum, Germany; 3 Department of Toxicology, Center for Research and Advanced Studies, CINVESTAV, Mexico City, Mexico; 4 Clinical Analysis Laboratory, Traumatology Hospital “Dr. Victorio De la Fuente Narvaez”, IMSS, Mexico City, Mexico; 5 Pneumology Service of the General Hospital, Medical Center La Raza, IMSS, Mexico City, Mexico; 6 Clinical Research, National Institute of Respiratory Diseases (INER), Mexico City, Mexico; 7 Integrative Immunology Laboratory, INER, Mexico City, Mexico; 8 Service Pathology, High Specialty Medical Unit (UMAE), Oncology Hospital, CMNSXXI, IMSS, Mexico City, Mexico; 9 Clinical Oncology Pneumology Service, INER, Mexico City, Mexico; 10 Research Department, INER, Mexico City, Mexico; 11 Department of Pathology, INER, Mexico City, Mexico; 12 Thorax Service, Oncology Hospital, High Specialty Medical Unit (UMAE), CMNSXXI, IMSS, Mexico City, Mexico; 13 Medical Oncology, Oncology Hospital, High Specialty Medical Unit (UMAE), CMNSXXI, IMSS, Mexico City, Mexico; 14 Service Chest Surgery, Hospital Cardiology, CMNSXXI, IMSS, Mexico City, Mexico; 15 Thoracic Surgery, Case Western Reserve University Hospitals of Cleveland, USA

**Keywords:** mesothelioma, mesothelin, calretinin, diagnostic marker, asbestos.

## Abstract

**Background:** Diagnosis of malignant pleural mesothelioma (MPM) remains a challenge, especially when resources in pathology are limited. The study aimed to evaluate cost-effective tumor markers to predict the probability of MPM in plasma samples in order to accelerate the diagnostic workup of the tissue of potential cases.

**Methods:** We conducted a case-control study stratified by gender, which included 75 incident cases with MPM from three Mexican hospitals and 240 controls frequency-matched by age and year of blood drawing. Plasma samples were obtained to determine mesothelin, calretinin, and thrombomodulin using enzyme-linked immunosorbent assays (ELISAs). We estimated the performance of the markers based on the area under the curve (AUC) and predicted the probability of an MPM diagnosis of a potential case based on the marker concentrations.

**Results:** Mesothelin and calretinin, but not thrombomodulin were significant predictors of a diagnosis of MPM with AUCs of 0.90 (95% CI: 0.85-0.95), 0.88 (95% CI: 0.82-0.94), and 0.51 (95% CI: 0.41-0.61) in males, respectively. For MPM diagnosis in men we estimated a true positive rate of 0.79 and a false positive rate of 0.11 for mesothelin. The corresponding figures for calretinin were 0.81 and 0.18, and for both markers combined 0.84 and 0.11, respectively.

**Conclusions:** We developed prediction models based on plasma concentrations of mesothelin and calretinin to estimate the probability of an MPM diagnosis. Both markers showed a good performance and could be used to accelerate the diagnostic workup of tissue samples in Mexico.

## Introduction

Malignant pleural mesothelioma (MPM) is an extremely lethal cancer strongly associated with exposure to asbestos. Asbestos is still an important commodity in global trade 1. About 50 countries banned the use of asbestos, but not yet Mexico, Colombia, Brazil, and many other countries worldwide. Even after cessation of exposure, the risk of developing an MPM is strongly elevated because of the long latency of this cancer 2. Efforts are under way to build international research networks in asbestos-related disease prevention 3. The Project 'MoMar' (Molecular Markers), for example, aims to identify and validate minimally-invasive tumor markers for the early detection of MPM with study groups from Mexico, Greece, Australia, and Germany 4, 5.

MPM diagnosis remains a challenge, and the prognosis is poor 6. In combination with imaging methods, tumor markers have been suggested to improve the diagnostic workup and to enhance survival 7, 8. Three markers have been selected in this study to evaluate their potential to assist the diagnostic workup. Mesothelin has been the most promising blood-based tumor marker so far 9, 10 and the well-established immunohistochemical marker calretinin was shown to be elevated in plasma samples of MPM patients 5, 11. Thrombomodulin, another immunohistochemical marker for MPM 12, was reasoned to be also a possible candidate for blood-based MPM detection. The involvement of these proteins in key processes of cancer development, such as proliferation and angiogenesis, render them also informative for therapeutic targets to improve the so far poor prognosis 13-15.

The Lancet Oncology Commission identified several obstacles to providing optimum cancer services in Latin America and the Caribbean 16. These limitations include insufficient activities for primary prevention, for example, the ban of asbestos. An update of this comprehensive evaluation addressed remaining challenges such as needs for a higher quality of the histopathological assessments 17. Due to the limited histopathological capacity, the diagnostic workup of tissue samples from potential cases with MPM can be strongly delayed in Mexico. Blood-based tumor markers that are fast, cost-efficient, and easy to determine may speed-up this process. Thus, the aim of our study was to predict the probability of a diagnosis of MPM based on the plasma levels of mesothelin, calretinin, and thrombomodulin in order to expedite the cases with the most likely MPM diagnosis to a histopathological examination of their tissue samples. Another - more long-term - goal is to find candidate markers for validation in prospective studies. Once validated these markers could be used for the early detection of MPM in screening programs in the future.

## Materials and Methods

### Study Population

A case-control study was conducted comparing tumor marker concentrations in blood samples from 75 incident MPM cases and 240 controls, which were enrolled with participation rates of 98% and 95%, respectively, in the Valley of Mexico from January 2012 to April 2015. All participants originated from and lived in urban areas. None of the participants were of indigenous origin but 96.4% were of Mestizo Mexican descent, while the remaining 3.6% had a more recent European or U.S. American background (first or second generation). Incident cases were recruited from the outpatient and inpatient services of three hospitals, who sought medical care with clinical suspicion of MPM. MPM diagnosis was confirmed by medical oncologists based on clinical examination, imaging tests (X-ray and chest computed tomography), biopsy, and immunohistochemistry. The panel of immunohistochemical biomarkers consisted of calretinin, cytokeratins (CK5/6), Wilms tumor protein (WT-1), vimentin, carcinoembryonic antigen (CEA), and thyroid transcription factor 1 (TTF-1/NKX2-1) 18. The subtype of MPM was classified according to WHO 19.

Cases were recruited among patients with health insurance at two hospitals from the Mexican Social Security Institute (IMSS) and among uninsured patients from Mexico's National Institute of Respiratory Diseases (INER), a hospital of Mexico's Ministry of Health. All hospitals are referral hospitals for respiratory diseases and associated cancers. Male controls (n=172) were matched by age and year of blood drawing to 63 cases. Female controls (n=68) were also matched by age and year of blood drawing to 12 women with epithelioid MPM at a higher ratio to improve the statistical power. Controls were selected from the National System of Beneficiaries database (SINDO) and from the IMSS' Information System on Severance Pensioners, Advanced and Old Age Workers 20. The controls were randomly selected from the respective database and invited by phone to voluntarily participate in the study. In addition, controls for INER cases were selected from facilities of the National Institute of Older People (INAPAM), which included day residences, comprehensive care centers, cultural centers, and clubs for elderly people.

An in-person interview and blood sampling were performed prior to the histopathological confirmation of the diagnosis of MPM. A questionnaire was applied by trained interviewers to assess socio-demographic information, a detailed occupational history, exposure to asbestos, smoking habits, medical history, and other data. Lifetime occupational exposure to asbestos was categorized as ever or never according to a previously published assessment 21. In brief, an industrial hygienist, who was unaware of the case-control status, estimated the exposure to asbestos according to the worker's job history and a list with information on industries importing asbestos or companies that manufactured asbestos fibers in various forms 22 along with recognized occupational activities and jobs with exposure to asbestos 23-28.

The research protocol was approved by IMSS' National Commission for Scientific Research and Ethics with registration number R-2011-785-069, and by INER's Committee on Science and Bioethics in Research with registration number C30-12. Prior to inclusion in the study, participants signed a letter of informed consent.

### Measurement of Tumor Markers in Plasma

Blood samples were drawn in three 6.0 ml tubes (EDTA vacutainers) and centrifuged at 2,250 g for 10 minutes in a laminar flow hood. Plasma and buffy coat were separated and stored at -80°C until analysis. Mesothelin and thrombomodulin ELISAs were performed at CINVESTAV in Mexico City, Mexico. For mesothelin determination, sandwich ELISAs were performed according to the manufacturer´s instructions (DY3265, R&D Systems, Minneapolis, MN). Thrombomodulin concentration was determined according to the manufacturer´s instructions (DY3947, R&D Systems).

Plasma samples were shipped to Germany under stringent frozen conditions and calretinin determination was performed utilizing a sandwich ELISA according to Raiko et al. 11 at the IPA in Bochum, Germany.

### Statistical Analysis

The distributions of the biomarker concentrations were presented by median and interquartile range (IQR). A relatively large number of calretinin concentrations were below the limit of detection (LOD). Therefore, affected percentiles were marked as being less (<) than the respective LOD (Table 1). For the calculations, we set values below LOD to two-thirds of LOD (2/3*LOD). Mesothelin and thrombomodulin concentrations between cases and controls were compared using the Wilcoxon rank-sum test. Because 72% of calretinin measurements were below LOD, the Peto-Prentice test was applied to compare cases and controls 29, 30.

Odds ratios (ORs) and 95% confidence intervals (CI) were calculated using unconditional logistic regression models to estimate the relative risk of a diagnosis of MPM based on the log-transformed concentrations of the tumor markers. These models were also used for estimating receiver operating characteristic (ROC) curves and the area under the curve (AUC) for the markers' sensitivity for varying values of specificity. Based on the performance of the markers we predicted the individual probability of a diagnosis of MPM for a male or female subject with the corresponding estimates of the true positive rate (TPR) and false positive rate (FPR) by these logistic regression models. Statistical analyses were performed using SAS software, version 9.4 (SAS Institute Inc., Cary, NC).

## Results

Table 1 depicts the characteristics of the cases and controls. Median age of MPM diagnosis was 64 years for males and 62 years for females. More women than men had never smoked (women: 66.7% in cases, 64.7% in controls; men: 33.3% in cases, 37.8% in controls). A large fraction of men was classified as ever exposed to asbestos at the workplace (93.7% in cases, 70.3% in controls). The corresponding figures in women were 33.3% and 17.7%, respectively. Epithelioid MPM was diagnosed for all twelve female cases and for 52 (82.5%) male cases.

Table 2 shows the distribution of the marker concentrations by case-control status in men and women. The concentrations of mesothelin and calretinin but not of thrombomodulin were statistically significant higher in cases than in controls, with distinct IQRs. In men, mesothelin median concentrations were 2.21 nmol/l in cases and 0.58 nmol/l in controls (p <0.0001). Similar concentrations were determined in women (2.00 nmol/l and 0.55 nmol/l, respectively, p <0.0001). The same pattern was observed for calretinin (men: 0.93 ng/ml in cases, <0.07 ng/ml in controls, p <0.0001; women: 0.86 ng/ml in cases, <0.22 ng/ml in controls, p <0.0037). No differences in thrombomodulin concentrations were observed between cases and controls (men: 2.57 vs. 2.30 ng/ml, p = 0.81; women: 2.25 vs. 2.05 ng/ml, p = 0.84). In addition, we show the distribution of the concentrations in subgroups stratified by age and exposure to asbestos.

Table 3 presents the ORs with 95% CI as estimates of the relative risk of an MPM per unit increase of the log-transformed concentrations of the markers. Whereas thrombomodulin was not associated with the development of MPM in men (OR=1.05, 95% CI 0.44-2.48) and women (OR=1.02, 95% CI 0.19-5.47), both mesothelin and calretinin were significant factors. The MPM risks per unit increase of the mesothelin and calretinin concentrations were 14.51 (95% CI 6.96-30.28) and 3.03 (95% CI 2.20-4.18) in men and 28.57 (95% CI 4.09-199.4) and 2.69 (95% CI 1.31-5.56) in women, respectively. In the multivariate analysis mesothelin appears to be more clearly associated with MPM risk than calretinin for males and females.

**Figure 1:**
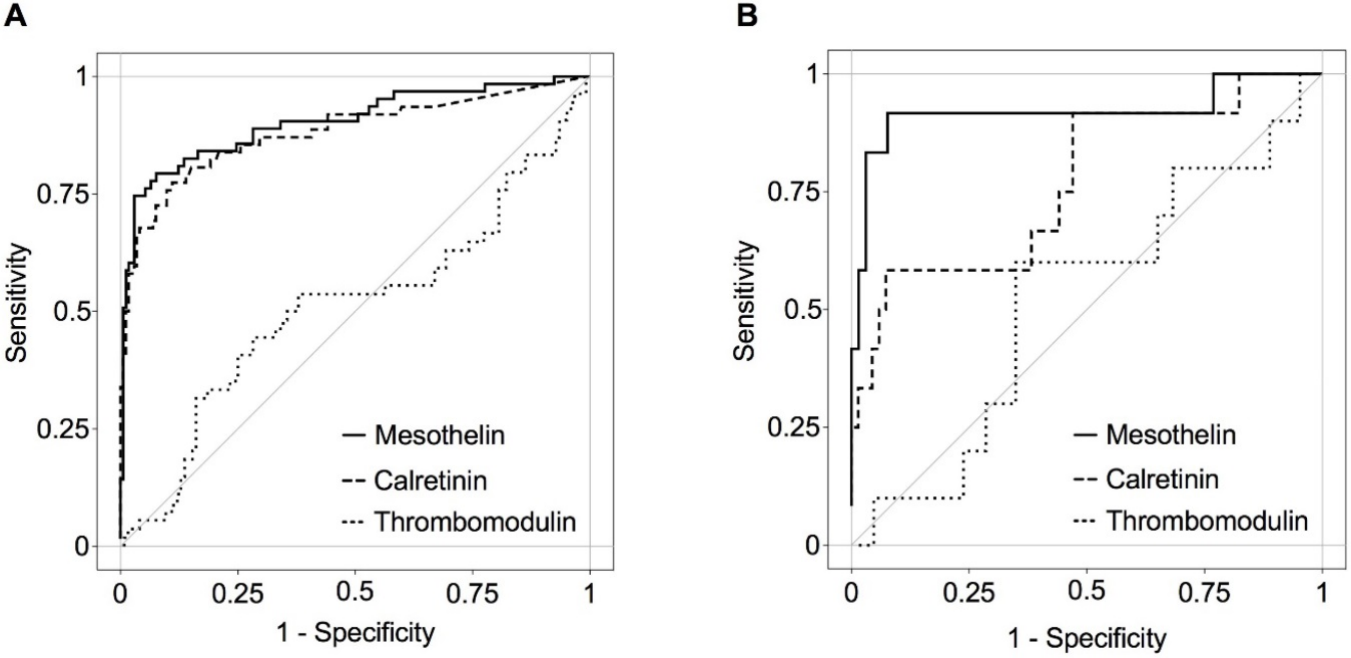
ROC curves of MPM biomarkers in incident cases and controls by gender (all CIs were 95%). A, Males: mesothelin (AUC=0.90, CI:0.85-0.95), calretinin (AUC=0.88, CI:0.82-0.94), and thrombomodulin (AUC=0.51, CI:0.41-0.61) B, Females: mesothelin (AUC=0.92, CI:0.79-1.00), calretinin (AUC=0.77, CI:0.61-0.93), and thrombomodulin (AUC=0.52, CI: 0.32-0.72).

Using ROC analyses, the calculated AUCs for the mesothelin concentrations were 0.90 (95% CI 0.85-0.95) in men and 0.92 (95% CI 0.79-1.00) in women. The AUCs for calretinin were slightly weaker, with 0.88 (95% CI 0.82-0.94) in men and 0.77 (95% CI 0.61-0.93) in women (Figure 1). Due to the low AUC, thrombomodulin was excluded from further analysis. Based on the plasma concentrations of mesothelin and calretinin we predicted the individual probability of an MPM in a male or female subject using the prediction formulas presented in Tables 4 and 5, respectively, resulting in estimated TPRs and FPRs for the whole study population stratified to probabilities ranging from 10% to 90%.

Utilizing mesothelin alone a pre-defined 30% probability for the diagnosis of MPM in an individual was associated with high TPRs in the study population (0.79 in men and 0.75 in women) and moderate FPRs (0.11 in men and 0.03 in women). For comparison, a high individual probability of 80% was associated with a moderate TPR of 0.43 and a low FPR of 0.01 in men and similar rates in women. An increase of the TPR, with stable FPR, could be achieved in men by including calretinin into the decision model (30% probability: TPR=0.84, 80% probability: TPR=0.53). Both markers are moderately correlated in plasma samples of cases (Kendall's tau correlation coefficient: 0.37, p <0.0001). The cut off values in Tables 4 and 5 can be used to apply the biomarkers to different tasks/issues, e.g., screening or optimizing histopathological diagnostic workup.

## Discussion

MPM is an aggressive cancer and difficult to diagnose, particularly at early stages. Reliable tumor markers could improve the diagnosis of MPM, including early stages 11, 31, 32. In some countries like Mexico, the resources for histopathological diagnosis of suspected cases are limited. MPM is also a rare disease, with a very low probability of an individual suffering from this type of cancer. Additional information on the blood concentrations of tumor markers can enhance an individual's probability. Here, we provided a prediction model for the probability of a diagnosis of MPM ranging from 10% to 90% in order to optimize the histopathological diagnostic workup of tissue samples.

Formulas given in Tables 4 and 5 can be used to calculate the probability of a subject for a diagnosis of MPM by gender for given plasma concentrations of mesothelin and calretinin. A high individual probability of a subject such as 90% is associated with a high tumor marker level. This high tumor marker concentration implies that the rates of true positives and of false positives at group level are lower. By contrast, a low individual probability of a subject such as 10% can be achieved at a lower tumor marker level. Hence, the corresponding rates of true positives and of false positives are higher.

For men, we observed a good individual performance of mesothelin as well as calretinin. A combination of both markers showed moderate improvement of the performance. For screening, one would want to achieve a high probability in order to keep the FPR low. For example, at 80% probability for a diagnosis of MPM mesothelin and calretinin showed a low rate of 1% and 0% false-positive decisions corresponding to 43% and 26% true-positive decisions, respectively. The combination of both markers resulted in 1% false-positive and 53% true-positive decisions. However, for diagnostic workup a lower probability can be accepted. Using a probability of 30%, mesothelin and calretinin showed 11% and 18% false-positive decisions with corresponding 79% and 81% true-positive decisions, respectively. The combination of both markers improved the performance to an acceptable 11% false-positive decisions and a relatively high rate of 84% true-positive decisions.

MPM is a very rare disease, especially in women where markers used to diagnose MPM may be also elevated in cases with cancer of the ovaries 33. Our prediction for female candidates with tissue samples waiting for histopathological diagnostic workup resulted in 3% false-positive decisions and 75% true-positive decisions for a probability of 30% for mesothelin alone. A combination with calretinin did not improve the performance of mesothelin. However, our study is of limited statistical power to estimate sound results for women, based on 12 cases only. Another, more general, limitation is the case-control design, where we cannot estimate the effect of age and gender as risk factors of developing an MPM due to the matching procedure.

Despite that a large number of candidate markers has been reported for MPM in the literature, mesothelin is still the best blood-based tumor marker available and the only one with FDA approval. It has a good performance to detect MPM with AUCs ranging between 0.72 and 0.93 34. Our results for mesothelin are in line with similar findings that MPM patients exhibit higher mesothelin concentrations in blood than healthy controls.

Calretinin is one of the best immunohistochemical markers for MPM diagnosis 35. This is the first study determining calretinin in plasma samples from Central America. Median calretinin concentrations in cases and controls were similar to those reported by Raiko et al. in samples from France and Germany and recently by Johnen et al. in samples from Australia and Germany 5, 11.

A few conditions may result in elevated marker concentrations and can thus lead to false-positive decisions. The association of pre-analytical variations with mesothelin were analyzed in several studies 36-38. Particularly renal failure was shown to have a strong effect on the mesothelin concentrations, resulting in false-positive tests 36. Within the framework of the MoMar study, we analyzed such pre-analytical variations in archived plasma samples from a prospective cohort of elderly subjects without malignant diseases 39. Knowledge of possible influencing factors could improve the performance of the markers.

It is common opinion that a panel of tumor markers may improve the performance of a single marker 31, 40, 41. However, many markers may change in parallel during carcinogenesis as could be shown in a statistical analysis of various markers applied to lung tissue 42. Depending on the chosen probability, the inclusion of calretinin into the prediction model with mesothelin led to an improvement, albeit moderate, of the marker performance. A major reason is the tight correlation between the plasma concentrations of the two proteins in cases. However, more sophisticated decision algorithms are needed to take advantage of the combination of both markers. A more promising approach to benefit from a panel may be to combine markers from different molecular levels 31, 41. We are currently investigating combinations of proteins with epigenetic as well as RNA markers. Initial results with mesothelin and the microRNA miR-103-3p look promising 43.

Many new MPM markers are emerging from small cross-sectional comparisons of cases and controls. Such a design suffers from methodological shortcomings 44. The most critical problem for application in symptom-free cohorts for the early detection of MPM is a potential overestimation of the sensitivity in cases with late-stage cancer. However, this is of minor concern in our current analysis. We took advantage of marker concentrations from patients with symptoms to predict an MPM diagnosis in order to accelerate the histopathological workup, facing limited resources in Mexico. In the future, however, it would also be desirable to provide markers to be used in surveillance programs for high-risk groups of asbestos-exposed workers to allow early detection of MPM. A timely treatment at early stages of tumor development should improve the prospects of survival. Before these markers can be used in symptom-free subjects, however, their performance to detect MPM earlier has to be validated using a prospective study design 41, 45. Because MPM is a rare disease, a sufficiently large cohort will be necessary 32, 46. Our study was conducted within the framework of MoMar, which is an international initiative to identify and validate markers for MPM. Within MoMar, an at-risk cohort of more than 2,700 former asbestos-exposed workers will be available for marker validation. Mesothelin and calretinin appear to be promising candidates for such a validation study. For translation into practice, marker assays not only need to have a good performance but also to be cost-effective, robust, and easy to apply. Because simple and robust ELISA-based assays are already available for the two markers, these requirements are basically met.

MPM represents a public health problem in Mexico. So far, the Mexican government has not followed WHO's recommendation to eliminate the use of asbestos. Since 1960, more than half a million tons had been imported from Canada, Russia, and Brazil 22. We estimated about 5,500 deaths from MPM in Mexico 1979 to 2015, taking into account an underreporting on death certificates 47. Just 10% of these cases were granted a general disability pension, but much fewer (ten cases) were recognized as an occupational disease in this period 20. Nearly all cases in this study had asbestos exposure but none was recognized as an occupational disease. It is important to receive a timely diagnosis, also for granting a compensation or pension. This is in contrast to Germany, where a large fraction of incident MPM cases with former exposure to asbestos have been recognized as occupational disease 48. Whereas the German industries provide the budget for surveillance, health care, and compensations offered by the German Social Accident Insurance, health care and pensions costs in Mexico are provided by IMSS and not by the companies causing health effects due to exposure to asbestos. A similar situation holds for INER, where patients are not insured 49.

## Conclusions

This is the first study of plasma markers for the prediction of MPM in a Mexican population with a high prevalence of exposure to asbestos to accelerate the diagnostic workup of the tissue for subjects with a high probability of such a diagnosis. It is urgent for the Mexican government to upgrade the resources in molecular pathology and to ban the use of asbestos. We developed a prediction model based on the plasma concentrations of mesothelin and calretinin. Both markers combined showed a good performance to estimate an individual's probability of an MPM diagnosis in conjunction with high TPRs and acceptable FPRs.

## Figures and Tables

**Table 1:** Description of the study population of cases with MPM and controls from Mexico

	Male				Female		
Characteristics	Cases N (%)	Controls N (%)	*P*-value^a^		Cases N (%)	Controls N (%)	*P*-value^a^
Total	63	172			12	68	
Age (years)							
Median (IQR)	64 (56-72)	62 (55-71)	0.59		62 (53-68)	61 (53-70)	0.82
Histologic diagnosis							
Epithelioid	52 (82.5)				12 (100)		
Sarcomatoid	3 (4.8)				-		
Other	7 (11.1)				-		
Not specified	1 (1.6)				-		
Smoking status			0.0003				-
Never smoker	21 (33.3)	65 (37.8)			8 (66.7)	44 (64.7)	
Former/current smoker	42 (66.7)	107 (62.2)			4 (33.3)	24 (35.3)	
Occupational exposure to asbestos			<0.0001				0.24
Never	4 (6.3)	51 (29.7)			8 (66.7)	56 (82.4)	
Ever	59 (93.7)	121 (70.3)			4 (33.3)	12 (17.7)	

^a^Continuous variables were compared using the Wilcoxon rank-sum test, categorical variables were compared using Fisher's exact test. IQR: interquartile range

**Table 2:** Distribution of biomarker concentrations in plasma samples from cases with MPM and controls from Mexico

		Cases				Controls			
		N	Median	IQR		N	Median	IQR	*P-*value^a^
*Mesothelin (nmol/L)*									
Males		63	2.21	1.37-3.93		170	0.58	0.40-0.87	<0.0001
Age	<70 years	41	2.04	1.28-3.77		120	0.52	0.38-0.76	
	≥70 years	22	2.75	2.14-3.93		50	0.76	0.48-1.02	
Occupational exposure to asbestos									
	Never	4	1.10	0.66-2.76		51	0.61	0.43-0.85	
	Ever	59	2.29	1.56-3.93		119	0.55	0.40-0.89	
								
Females		12	2.00	1.31-5.40		65	0.55	0.41-0.79	<0.0001
Age	<70 years	9	1.46	1.28-3.24		48	0.55	0.41-0.78	
	≥70 years	3	6.20	2.13-9.90		17	0.59	0.31-0.95	
Occupational exposure to asbestos									
	Never	8	1.80	1.29-5.40		53	0.55	0.38-0.79	
	Ever	4	2.56	1.57-5.01		12	0.55	0.45-0.87	
									
*Calretinin* (*ng/mL*)									
Males		62	0.93	<0.45-2.18		172	<0.07	<0.01-<0.22	<0.0001
Age	<70 years	40	1.00	<0.31-2.50		120	<0.08	<0.01-<0.23	
	≥70 years	22	0.85	0.58-1.98		52	<0.06	<0.01-<0.22	
Occupational exposure to asbestos									
	Never	4	1.41	<0.74-2.16		51	<0.06	<0.01-<0.20	
	Ever	58	0.86	<0.45-2.18		121	<0.08	<0.01-<0.24	
									
Females		12	0.86	<0.25-1.33		68	<0.22	<0.10-0.48	0.0037
Age	<70 years	9	<0.35	<0.24-1.12		50	<0.22	<0.12-0.48	
	≥70 years	3	3.09	0.97-4.98		18	<0.18	<0.03-<0.46	
Occupational exposure to asbestos									
	Never	8	1.04	<0.55-2.16		56	<0.22	<0.10-0.48	
	Ever	4	<0.25	<0.13-<0.85		12	<0.27	<0.08-<0.47	
									
*Thrombomodulin* (*ng/mL*)									
Males		54	2.57	1.71-3.18		124	2.30	1.84-2.85	0.81
Age	<70 years	35	2.01	1.66-2.90		96	2.24	1.79-2.76	
	≥70 years	19	3.04	1.97-3.41		28	2.79	2.19-3.38	
Occupational exposure to asbestos									
	Never	3	2.84	1.72-3.17		29	2.22	1.70-2.87	
	Ever	51	2.53	1.67-3.25		95	2.31	1.86-2.83	
									
Females		10	2.25	1.77-2.43		63	2.05	1.62-2.72	0.84
Age	<70 years	8	2.07	1.59-2.36		47	2.01	1.58-2.51	
	≥70 years	2	2.50	2.27-2.73		16	2.26	2.06-3.14	
Occupational exposure to asbestos									
	Never	6	2.10	1.77-2.30		51	2.05	1.66-2.72	
	Ever	4	2.33	1.82-3.19		12	2.09	1.26-2.66	

^a^Mesothelin and thrombomodulin concentrations were compared using the Wilcoxon rank-sum test, calretinin concentrations were compared using the Peto-Prentice test. IQR: interquartile range; '<' indicates percentiles below the limit of detection (LOD)

**Table 3:** Odds ratios from logistic regression analyses as estimates of the relative risk for an MPM based on the plasma concentrations in cases and controls

Study group	Characteristics	Intercept	Coefficient	OR (95% CI)
Males	Models			
	*Univariate*			
	ln(Mesothelin)[nmol/L]	-1.15	2.68	14.51 (6.96-30.28)
	ln(Calretinin)[ng/mL]	0.55	1.11	3.03 (2.20-4.18)
	ln(Thrombomodulin) [ng/mL]	-0.87	0.04	1.05 (0.44-2.48)
				
	*Multivariate*			
	ln(Mesothelin)[nmol/L]	-0.17	2.11	8.26 (3.77-18.10)
	ln(Calretinin)[ng/mL]		0.59	1.80 (1.31-2.47)
				
Females	Models			
	*Univariate*			
	ln(Mesothelin)[nmol/L]	-1.77	3.35	28.57 (4.09-199.4)
	ln(Calretinin)[ng/mL]	-0.72	0.99	2.69 (1.31-5.56)
	ln(Thrombomodulin)[ng/mL]	-1.86	0.02	1.02 (0.19-5.47)
				
	*Multivariate*			
	ln(Mesothelin)[nmol/L]	-1.36	3.08	21.86 (3.13-152.51)
	ln(Calretinin)[ng/mL]		0.33	1.39 (0.67-2.88)

OR: odds ratio; CI: confidence interval; ln: natural logarithm

**Table 4:** Performance of mesothelin and calretinin in men and the probability of a diagnosis of MPM, conditional on the observed biomarker concentrations

Probability %	Males											
	Model 1				Model 2				Model 3			
	Cut off Mesothelin [nmol/L]	TPR	FPR		Cut off Calretinin [ng/mL]	TPR	FPR		Cut off Mesothelin [nmol/L]	Cut off Calretinin [ng/mL]	TPR	FPR
90	3.48	0.33	0.01		4.47	0.10	0		3.66	0.85	0.37	0.01
80	2.62	0.43	0.01		2.18	0.26	0		2.15	1	0.53	0.01
70	2.09	0.57	0.01		1.36	0.40	0.01		1.08	4.25	0.63	0.01
60	1.81	0.63	0.03		0.87	0.52	0.02		1.46	0.79	0.73	0.03
50	1.56	0.73	0.03		0.62	0.68	0.05		0.74	3.82	0.76	0.05
40	1.31	0.78	0.06		0.41	0.76	0.10		0.97	0.74	0.84	0.07
30	1.12	0.79	0.11		0.29	0.81	0.18		1.01	0.33	0.84	0.11
20	0.92	0.84	0.22		0.18	0.87	0.34		0.65	0.58	0.85	0.14
10	0.68	0.90	0.40		0.09	0.92	0.49		1.31	0.01	0.92	0.33

Probability was used to estimate TPR and FPR: *Probability* = 1/(1+e^(-φ)). Logistic regression models: (1) with log-mesothelin as predictor, φ = exp [-1.15 + 2.68 * ln(*mesothelin*)]; (2) with log-calretinin as predictor, φ = exp [0.55 + 1.11 * ln(*calretinin*)]; (3) with log-mesothelin and log-calretinin, φ = exp [-0.17 + 2.11 * ln(*mesothelin*) + 0.59 * ln(*calretinin*)]. TPR: true positive rate; FPR: false positive rate

**Table 5:** Performance of mesothelin and calretinin in women and the probability of a diagnosis of MPM, conditional on the observed biomarker concentrations

Probability %	Females											
	Model 1				Model 2				Model 3			
	Cut off Mesothelin [nmol/L]	TPR	FPR		Cut off Calretinin [ng/mL]	TPR	FPR		Cut off Mesothelin [nmol/L]	Cut off Calretinin [ng/mL]	TPR	FPR
90	3.24	0.42	0		-	0	0		-	-	0.33	0
80	-	0.42	0		-	0	0		2.13	3.09	0.50	0
70	-	0.42	0.02		4.98	0.08	0		-	-	0.50	0.02
60	1.87	0.58	0.02		3.09	0.17	0		-	-	0.58	0.02
50	-	0.58	0.02		-	0.17	0		-	-	0.58	0.02
40	1.48	0.58	0.03		1.44	0.25	0		1.35	1.23	0.75	0.02
30	1.35	0.75	0.03		0.97	0.50	0.06		1.21	1.12	0.75	0.05
20	1.14	0.92	0.12		0.49	0.58	0.24		1.05	0.72	0.83	0.09
10	0.90	0.92	0.20		0.23	0.92	0.49		0.90	0.23	0.92	0.20

Probability was used to estimate TPR and FPR: *Probability* = 1/(1+e^(-φ)). Because of the small number of female cases (12), not for all set probabilities corresponding marker concentrations were available. Logistic regression models: (1) with log-mesothelin as predictor, φ = exp [-1.77 + 3.35 * ln(*mesothelin*)]; (2) with log-calretinin as predictor, φ = exp [-0.72 + 0.99 * ln(*calretinin*)]; (3) with log-mesothelin and log-calretinin, φ = exp [-1.36 + 3.08 * ln(*mesothelin*) + 0.33 * ln(*calretinin*)]. TPR: true positive rate; FPR: false positive rate

## Abbreviations

AUC: area under the curve
CI: confidence interval
ELISA: enzyme-linked immunosorbent assay
FPR: false positive rate
IQR: interquartile range
LOD: limit of detection
MPM: malignant pleural mesothelioma
OR: odds ratio
ROC: receiver operating characteristic
TPR: true positive rate.
